# Drugs Interfering with Muscarinic Acetylcholine Receptors and Their Effects on Place Navigation

**DOI:** 10.3389/fpsyt.2017.00215

**Published:** 2017-11-09

**Authors:** Jan Svoboda, Anna Popelikova, Ales Stuchlik

**Affiliations:** ^1^Institute of Physiology of the Czech Academy of Sciences, Prague, Czechia

**Keywords:** scopolamine, biperiden, acetylcholine, receptor, behavior, learning, memory, rodents

## Abstract

Muscarinic acetylcholine receptors (mAChRs) have been found to regulate many diverse functions, ranging from motivation and feeding to spatial navigation, an important and widely studied type of cognitive behavior. Systemic administration of non-selective antagonists of mAChRs, such as scopolamine or atropine, have been found to have adverse effects on a vast majority of place navigation tasks. However, many of these results may be potentially confounded by disruptions of functions other than spatial learning and memory. Although studies with selective antimuscarinics point to mutually opposite effects of M1 and M2 receptors, their particular contribution to spatial cognition is still poorly understood, partly due to a lack of truly selective agents. Furthermore, constitutive knock-outs do not always support results from selective antagonists. For modeling impaired spatial cognition, the scopolamine-induced amnesia model still maintains some limited validity, but there is an apparent need for more targeted approaches such as local intracerebral administration of antagonists, as well as novel techniques such as optogenetics focused on cholinergic neurons and chemogenetics aimed at cells expressing metabotropic mAChRs.

## Introduction

Acetylcholine (ACh) is one of the major neurotransmitters and modulators of the nervous system. Its receptors are abundantly expressed in a wide variety of tissues, from neuromuscular junctions and parasympathetic system to cortical regions involved in cognitive functions such as learning and memory (1). The cholinergic system has been shown to play an important role in processes such as circadian rhythmicity (2), addiction (3), motivation, pain, and reward (1), as well as cognitive flexibility (4), perceptual memory (5), spatial learning (6), and many more. It comes as no surprise that abnormalities in the function of the cholinergic system and its components underlie a multitude of pathologies, such as Parkinson’s disease (7), Alzheimer’s disease (AD) (8), schizophrenia, bipolar disorder (9, 10), and depression (11). For these reasons, the cholinergic system has been extensively studied in recent years; however, many mechanisms of its function still remain unclear.

## ACh System in the Brain

There are two main types of ACh receptors, named historically after their naturally occurring alkaloid agonists: (1) nicotinic receptors (nAChRs), a family of ionotropic receptors which act as ligand-gated cation channels and (2) muscarinic Ach receptors (mAChRs), a metabotropic G-protein-coupled receptor (GPCRs) family whose activation may trigger various responses depending on the specific subtype and context of the signal (8). Nicotinic ACh receptors are named after nicotine, their prototypical agonist. Probably the most famous antagonist of the nAChRs is D-tubocurarine, a compound isolated from the curare poisons (12, 13). mAChRs are named after muscarine, a toxic alkaloid synthesized in the toadstool *Amanita muscaria*. Possibly the best known antagonist of the mAChRs is atropine, found in the deadly nightshade (*Atropa belladonna*) (14).

### Nicotinic Receptors

Despite being best known for their involvement in signal transduction at neuromuscular junctions, these receptors are also expressed throughout the central nervous system (CNS). As mentioned above, nicotinic ACh receptors are ionotropic, i.e., ligand-gated cation channels, whose activation by an agonist evokes a flux of K^+^, Ca^2+^, and Na^+^ ions (however not all subtypes of nAChRs are permeable for Na^+^), which in turn triggers mechanisms of Ca^2+^ signaling (1). These receptors typically comprise five subunits: either a homomeric combination of α subunits (for example α7) or a heteromeric combination of α(1–10), β(1–4), δ, and ε subunits. The specific combination of these subunits results in different pharmacological properties of the individual subtypes, such as ion selectivity and ligand affinity (14). The most common nAChR subtypes found in the brain are α7 and α4β2 receptors. Located at both pre- and postsynaptic sites, they play a pivotal role in various processes, such as learning and cognition (15), decision-making (9), and regulation of the postnatal development of the visual cortex (16). Thus, nicotinic ACh receptors constituted of specific subunits appear to be suitable pharmacological target for cognitive enhancement.

### Muscarinic Receptors

Muscarinic receptors are abundantly expressed throughout the brain; however, they are also found in various other tissues in the body, such as the heart (17, 18), the bladder and pulmonary system (19), and the intestine (20). As mentioned above, mAChRs do not serve as cation channels like nicotinic receptors, but instead are coupled with G-proteins, which transmit signals into the cell by affecting the activity of certain enzymes (such as the adenylyl cyclase, phospholipase C, etc.) (14, 21).

Five subtypes of mAChRs have been described, M1–M5. They differ in their level of expression in various parts of the body and the signal cascades they trigger after binding an agonist. Located mostly postsynaptically, the M1, M3, and M5 receptors (sometimes referred to as “M1-like” receptors) activate phospholipase C *via* G_q/11_ protein, thus inducing a calcium influx into the cell. M2 and M4 on the other hand (the “M2-like” group), when activated lower the level of cyclic adenosine mono-phosphate in the cell by G_o/i_ protein-mediated inhibition of adenylyl cyclase. They are found both pre- and postsynaptically (8, 21, 22).

The outputs of signaling through specific cholinergic receptor subtypes may vary tremendously depending on the subtype of the receptors and their pre- or postsynaptic localization. The specific tissue and the type of the cell that expresses the receptors is also of major importance, as well as the metabolic state of the neuron at the precise time of receiving the signal, i.e., a cell with high intracellular levels of calcium may react differently to a signal than one with low intracellular concentrations of calcium. To further complicate any predictions of outcomes of cholinergic signaling and behavioral analysis, many neurons corelease ACh and glutamate, or ACh and gamma-aminobutyric acid (4, 21).

One of the major characteristics of the molecular structure of mAChRs is the evolutionarily highly conserved orthosteric ACh binding site, with a key asparagine residue (Asn105). This results in great difficulty when developing direct agonists and antagonists selective for a specific receptor subtype, and non-selective agents such as scopolamine, an antiemetic drug, and 3-iodothyronamine are widely used in research on memory impairment (23–25). Researchers have rather focused on developing compounds acting as allosteric ant/agonists and positive allosteric modulators (8, 26).

#### M1 mAChR

The M1 receptor is considered to be the most abundant subtype (50–60% of all mAChRs) of mAChRs in the brain. It plays an essential role in many cognitive functions such as learning and memory, and thus has become a target of research focusing on developing therapeutics for neurodegenerative diseases (8, 10, 27). For example, Ragozzino et al. (28) reported an enhancing effect of CDD-0102A, a partial M1 agonist, on working memory and strategy changing in rats. The compound improved the rats’ performance in a spontaneous alteration task (designed to test working memory) and, under changed circumstances, their ability to deem a previously useful strategy irrelevant and to find and retain a new one. They demonstrated the involvement of M1 receptors in these processes, and further suggested the use of CDD-0102A as a potential therapeutic agent for disorders such as AD and schizophrenia, emphasizing its enhancing influence and the lack of observed adverse effects (28). The M1 receptor is also expressed in other tissues than the brain; for example it has been shown to participate in the regulation of non-quantal ACh release in neuromuscular junctions (29).

## Antimuscarinic Drugs

Due to the diverse expression and functions of AChR in the brain, compounds affecting the cholinergic neurotransmission are employed in the treatment of a wide range of conditions and diseases. They are generally used for antiparkinsonian treatments, specifically targeting extrapyramidal symptoms such as rigidity, tremors, and bradykinesia. For example, it is generally accepted that an imbalance of cholinergic and dopaminergic transmission in the brain is one of the mechanisms underlying or accompanying schizophrenia, particularly in the negative symptoms and cognitive impairment. Anticholinergic drugs are often prescribed along with antipsychotics to alleviate their unwanted side effects. However, their usage has often been questioned as they themselves cause a range of side-effects, such as cognitive impairment, tardive dyskinesia, blurred vision, dry mouth, problems with urinary retention, psychosis, addiction, and many more (30–32). To give an example, Veselinović et al. (33) investigated the effect of the administration of anticholinergics on cognition in untreated patients with schizophrenia and healthy control subjects. Their results showed a marked impairment in both groups, which was however more pronounced in the schizophrenia patients, thus again casting doubt on the suitability of these drugs in the treatment of schizophrenia (33).

Interestingly, some antimuscarinic agents (namely scopolamine) also appear to possess antidepressant qualities, especially in treatment of those patients who are unresponsive to the standard therapy. Witkin et al. (11) reported that these antidepressant effects might be mediated specifically by the blockage of the M1 and M2 receptors (11).

The general consensus is that anticholinergics disrupt acquisition learning and long-term memory processing. As such, these compounds are often employed for inducing memory and cognitive impairments in laboratory animals in order to model pathological states observed in human diseases such as schizophrenia, AD and other dementias (5). Despite its popularity, such an approach has received a lot of criticism. For example, antimuscarinic agents provide only a limited predictive and face axes of validity, but low construct validity in AD research. Furthermore, it is sometimes very difficult to tease apart effects on memory and attention, or procedural deficits in general, that are separable from the cognitive deficits in many navigational tasks (34).

### Mechanisms of Action

As mentioned above, the ACh binding site is evolutionarily highly conserved across all five mAChR subtypes, which in turn complicates the search for subtype-selective ligands. However, there is an abundance of allosteric sites that facilitate receptor activity modulation and are specific for each receptor subtype. These have enabled the development of highly selective compounds (8).

Orthosteric subtype-selective agents are scarce, though some may be found; for example, a recent study reported a novel compound PCS1055 that exhibits high selectivity for the M4 receptor (35). Also, some ligands have been shown to bind at the orthosteric site as well as one of the allosteric sites, thus achieving relatively high selectivity for a specific mAChR subtype. An example may be seen in the work of Jakubík et al. (36), where the mechanism of action of the M2-selective antagonist methoctramine was put under scrutiny. The authors reported that methoctramine binds with high affinity to the orthosteric site and at the same time interacts with lower affinity with an allosteric site at the second and third extracellular loops. Interestingly, in the presence of another orthosteric-binding ligand (such as N-methyl-scopolamine), methoctramine may still bind to the allosteric site, thus preventing the other ligand from dissociating from the receptor. This antagonist occasionally binds the M3 receptor as well, but with a much lower affinity due to the lack of the allosteric site found on M2 (36). Also, the time that antagonists take to bind to the receptor has been shown to be of crucial importance for the efficacy of receptor blockage. For example, due to its relatively slow binding, tiotropium seems less effective at blocking the M3AChR (37).

As to the effects of antimuscarinic drugs on the organism, these naturally depend on the means and site of administration (which determines where the agent exerts its influence, such as the brain following an intracerebroventricular injection or the heart after a systemic application of a drug unable to cross the blood–brain barrier). Thus, as the M1 and M4 receptors are abundantly expressed in parts of the brain affected in neurodegenerative diseases such as AD, it seems probable—and has been repeatedly reported—that stimulating cholinergic transmission *via* these receptors should enhance cognitive abilities, learning and memory, whereas blocking it would result in cognitive impairment (26).

### Clinical Potential of Antimuscarinic Drugs

In spite of the risk of various undesirable side-effects such as cognitive impairment, dry mouth, or even psychosis and addiction, if dosed with care, antimuscarinic drugs provide therapeutic effects in a number of conditions. For illustration, aclidinium and tiotropium are often prescribed in the treatment of chronic pulmonary disease, as well as asthma, overactive bladder, and irritable bowel syndrome (38–41).

Quite recently, scopolamine, a non-selective antagonist capable of crossing the blood–brain barrier, has been found to exhibit antidepressant properties (mediated probably by its binding to M1 and M2 receptors), even in patients unresponsive to standard therapy (11). This has proven beneficial not only to patients with major depressive disorder but also to those suffering from bipolar disorder (42). In addition, scopolamine is also used as an antiemetic, for example in treating postoperational nausea (23).

As mentioned previously, mAChR antagonists (e.g., biperiden, trihexyphenidyl) are also employed as prophylaxis and/or for the treatment of side-effects of antipsychotics prescribed in diseases such as schizophrenia. However, this method is currently on the decline due to the multitude of unwanted side-effects of the anticholinergic treatment (30, 33).

Biperiden, amongst other antimuscarinics, also acts as an antiparkinsonian agent and is thus sometimes prescribed to patients with Parkinson’s disease, as well as other diseases manifesting with parkinsonian symptoms. However, even here the risks of addiction and detrimental side-effects still remain (43, 44).

Quite surprisingly, given the amount of criticism regarding the cognitive side-effects of muscarinic antagonists, a recent study investigating the properties of a new potential treatment for AD reported M1-antagonism for these agents. The tested drug candidate was developed using a newly proposed approach to treating multifactorial diseases such as AD, which aims to hit multiple therapeutic targets with a single drug comprising a series of compounds, in this case combining 7-methoxytacrine and memantine. As the results of other tests (such as successful prevention of β-amyloid fibrillization, AChE inhibition, etc.) looked rather promising, the authors recommended the novel compound as a potential treatment, claiming that the observed M1-antagonism did not seem to exhibit noticeable effects (45). It is conceivable that muscarinic antagonism can act beneficially when it is a part of a broader spectrum of mechanisms of action.

### Biperiden As a Prototype Drug

Biperiden hydrochloride (or lactate) is an established M1-receptor selective antagonist. Approved for human usage and sold under the brand name of Akineton, it is prescribed for Parkinsonism (to improve motor abilities such as gait and tremor) and occasionally to suppress the side-effects of neuroleptics.

Apart from clinical practice, biperiden is also used in research as a cognitive impairer (46, 47). Biperiden has been shown to cross the blood–brain barrier without difficulties, thus enabling a simple administration of the drug, such as using intraperitoneal or subcutaneous injections (s.c.). The tissue distribution (*V*_d_) for biperiden has been reported to be relatively high: with a brain to plasma ratio of up to 7–12 (44). The uptake of the drug by the tissues is quite rapid, possibly also due to its substantial transport into lysosomes (48). This makes biperiden a useful candidate as a specific drug, contrarily to scopolamine or atropine.

## Place Navigation

To increase their chances of survival, including successful foraging for food and other resources, as well as finding their nest or burrow, animals employ a variety of spatial navigation strategies. In principle, such strategies can be based on idiothesis or allothesis (or a combination of both). In the first case, an individual finds its way based on the information from vestibular receptors, muscle proprioceptors and tendon receptors complemented with efference copies of motor commands and/or optic and haptic flow, whereas in the second case, the spatial representation is established upon external cues (49). Three navigation strategies may be used to reach a goal:

(1) *a praxis strategy*, when an animal follows a set of learned, usually stereotypic movements that lead to a known goal,
(2) *a taxon strategy*, when the goal is clearly visible from a distance or marked by other cues,
(3) *a spatial strategy or mapping*, when long-distance external cues become the spatial reference points, as the goal cannot be located otherwise (by sight or smell) (50–52).

To illustrate, a man waking up at night and finding his way to the bathroom in the dark employs a *praxis* strategy; he knows it takes approximately four steps to the door of the room and then he has to turn right in the hallway and walk five more steps. A *taxon* strategy is used for example by a man approaching a bank—a large conspicuous building bearing an easy-to-see “Bank” sign. Finally, the mapping strategy focuses on finding the correct configuration of distal external cues, such as a man searching for a buried treasure (after his unsuccessful trip to the bank): e.g., he has to stand at a place with the big pine tree to his left, the strangely shaped mountain on the horizon behind him, and the lake a short distance in front of him.

Spatial navigation is based on the so-called *place coding* (53). The key structure of the brain involved in these processes is generally thought to be the hippocampus (more specifically its dorsal part); however, other parts of the brain play important roles as well. The neuronal substrate consists of place cells, large hippocampal pyramidal neurons with characteristic complex spikes that fire only in a specific part (or parts) of a given environment [the so-called *firing fields* or *place fields* (54); for review see Ref. (55)]. Interestingly, their structural organization in the brain is not topological, i.e., it does not reflect the outside world. Groups of these cells constitute *ensembles*, which serve as representations of the environment (56). Apart from these, there are grid cells, located in the entorhinal cortex (57). The spatial pattern of their firing fields resembles a hexagonal grid. And the final type is represented by head direction cells, found in the Papez circuit, and whose activity is dependent on the inclination or direction of an individual’s head (49, 53, 58–60). The specific roles and mechanisms of function of these cells are not yet fully understood. A recent study has proposed a model for spatial navigation based on cooperation between place cells and grid cells, in which place cells are responsible mainly for locating a goal, whereas grid cells are in charge of directing an individual toward that goal (60).

Other important aspects of effective spatial navigation are sets of spatial stimuli that yield so-called frames of reference. An individual often needs to distinguish and correctly assess conflicting information from several of these frames to solve a task. An example of a behavioral test specifically assessing this ability is active place avoidance (see Active Place Avoidance Tasks). The hippocampus has been shown to be the structure responsible for organizing this spatial information into representations correctly corresponding to the outside world (61–64). Behavioral tests based on spatial navigation are largely used by researchers in studying certain types of memory.

## Antimuscarinic Agents in Spatial Tasks

### Morris Water Maze (MWM)

#### Non-Specific Antagonists

Scopolamine is possibly one of the most frequently used antimuscarinic agents in the MWM. In spite of becoming something of a “gold standard” in research of cognitive impairment, its validity as a model has often been questioned because of its considerable side effects. As it lacks selectivity for any of the subtypes of mAChRs, apart from memory and cognition it also affects the sensorimotor functions of the treated subjects, thus sometimes compromising the results of the behavioral tests (65). However, Robinson et al. (66) reported impaired performance in the MWM in both rats and mice following scopolamine administration at a dose that exhibited no effect on visual acuity. This was studied in a variant of the MWM task specially adjusted to test for compromised visual perception, in which the animals were required to discriminate between two marginally differing cards in order to successfully find the hidden platform (66). A lack of effect on performance in a mainly vision-reliant task (the visible platform variant of the MWM) was also reported by Entlerova et al. (67) in their study focusing on a comparison of two commonly used rat strains (Wistar and Long-Evans) and their performance and sensitivity to anticholinergic blockade in the MWM and active place avoidance. Following scopolamine treatment, they found no marked differences in the MWM between the two strains, whereas in active place avoidance the Wistar rats exhibited significantly worse performance than the Long-Evans group, suggesting a higher sensitivity to scopolamine in the Wistar strain (67).

Furthermore, von Linstow Roloff et al. (68) set out to investigate whether the poor performance of scopolamine-treated rats in the MWM is in any part due to an effect on memory processes, or whether it is just the result of compromised sensorimotor abilities. In a series of experiments consisting of acquisition tasks combined with both spatial and non-spatial pretraining, as well as delayed-match-to-position (DMP) and a variant of the DMP with an on-demand platform [also called the Atlantis platform (69, 70)], they were able to show that although scopolamine undoubtedly causes side-effects leading to altered swimming speeds and higher levels of thigmotaxis, these can be eliminated by extensive spatial pretraining. In such a case however, scopolamine-treated animals still perform more poorly than controls, thus confirming that scopolamine does indeed affect spatial memory. In the Atlantis platform paradigm, the researchers were able to discriminate between the effects on procedural and spatial memory: scopolamine was found to impair the latter (68).

Navigating to a submerged platform requires a mapping strategy. As reviewed in Ref. (6), scopolamine disrupts forming a memory for platform location that is held constant across days (reference memory) or changes daily (working memory). When directly compared, working memory seems to be affected more than reference memory (71). Compromised navigation in the water maze can be explained in terms of the inaccurate positional information of place cells. Intraventricular or intrahippocampal infusions of scopolamine increase the firing of place cells outside of the usual place cell firing field of the neuron, leading to lesser place specificity (72, 73). Scopolamine seems to also affect other correlates of spatial memory. Its systemic administration flattens the typically robust positive correlation between running speed and theta frequency (74) and reduces spatial tuning of the grid cells (75). However, at least in the entorhinal cortex, scopolamine does not alter the tuning of head direction cells (75).

Water maze studies are able to provide some evidence regarding how scopolamine specifically affects particular stages of memory processing. There is general agreement on its effects on memory encoding [reviewed in Ref. (76)], while reports on consolidation or recall are mixed. Most studies report no or little effect on consolidation or recall (6, 77, 78) but a recent investigation demonstrated that systemic scopolamine administration in mice had a detrimental effect on the retrieval of platform location (79).

Scopolamine-induced cognitive impairment has also been shown to possess good validity as a translational model in research. Laczó et al. (80) compared the effects of scopolamine administration (as well as its coadministration with donepezil, an AChE inhibitor) in rats and humans in the MWM and the Hidden Goal Task, an analog of the water maze fit for use in humans. The authors reported successful validation of the tasks and scopolamine, as no significant differences were found between the human volunteers and the animals. Donepezil was shown to exhibit some ameliorative effect; however, this was not clear in all cases (80).

Although mostly of an older date, studies examining the effects of other antimuscarinic agents may also be found. In one such report by Sutherland et al. (52) focused on atropine, atropine sulfate-treated rats were found to lack the ability to employ spatial mapping as means of learning the location of the hidden platform, thus turning to a combination of taxon and praxis strategies (i.e., not remembering the position of the platform but instead rather a way of finding it). No such deficit was observed in control animals and a group treated with atropine methylnitrate (a substance acting solely in the periphery as it is unable to cross the blood–brain barrier), hence confirming the hypothesis that the central cholinergic system underlies spatial mapping strategies (52). It has also been proposed that atropine may interfere with the ability to inhibit non-efficient spatial strategies that appear initially during water maze acquisition (81).

The use of the MWM also occurred in a report assessing the properties of 3-quinuclidinyl benzilate (QNB), a non-selective muscarinic antagonist that has been proposed as a potential agent for modeling cognitive deficits in rats. The study showed a significant detrimental effect of QNB on acquisition in the MWM, whereas no impairment was found in memory consolidation and retrieval. Apart from hyperlocomotion leading to higher swimming speeds, the authors observed no adverse side effects of QNB on vision and sensorimotor functions (82). A study on oxybutynin, an antagonist of M1, M2, and M3 receptors, further confirmed that non-selective antagonists exert detrimental effects on acquisition in the MWM (83).

#### M1-Like Family mAChR Antagonists

Due to their abundance, it has been suggested that the effects of non-selective antagonists may be exerted primarily through M1 receptors. However, it turned out that attempts to silence M1 receptors functioning have provided mixed results. Pirenzepine, a selective M1 antagonist, was evaluated in the studies of Hagan et al. (84) and Hunter and Roberts (85). Although less potent than scopolamine, it was nevertheless shown to impair spatial navigation in the MWM while preserving the taxon strategy (navigation to a visible platform). However, one of the major drawbacks of this drug is its inability to cross the blood–brain barrier, thus requiring intraventricular administration (84, 85). In contrast to that line of evidence, mice lacking M1 receptors display unimpaired performance in a water maze in spite of general hyperactivity (86). Furthermore, systemic administration of imidafecin, a selective M1 and M3 antagonist, appeared to have no significant effect on navigation in a water maze (83). These results therefore questioned the exclusive role of M1 receptors in scopolamine-induced deficits in water maze navigation. In an attempt to explain this discrepancy, Bubser et al. concluded that M1 receptors seem to play a more significant role in mPFC-mediated tasks than in hippocampus-dependent tasks (87).

#### M2-Like Family Antagonists

An exception to the “rule” of muscarinic antagonists having detrimental effects on learning and memory are compounds selective for receptors expressed presynaptically (such as M2), which by blocking the presynaptically mediated inhibition of ACh release actually help to increase the levels of ACh in the synapse, and thus also cholinergic transmission (88, 89). For example, BIBN-99, a selective M2 antagonist, has been shown to improve the performance of aged rats in the MWM (88). Involvement of the M4 receptor in a water maze was assessed using M4 receptor knock-out mice. Despite elevated locomotion observed in the open field, knock-out mice displayed both unaltered acquisition and preference to a target location in probe trials in the water maze (90). It can be generally concluded that M2-like family muscarinic antagonists have weaker and sometimes even positive effects on place navigation tasks due to the different neuronal localization of respective receptors and the *de facto* different mechanistic mode of action, resulting in specific behavioral outcomes.

Results obtained with the MWM generally support the conclusion that antimuscarinic drugs adversely affect place navigation. On the other hand, this task also points to a number of non-cognitive confounding variables in the effects of antimuscarinic agents in place learning and memory. Importantly, muscarinic antagonists specific for particular receptor subtypes have been found to have only partial advantages over non-specific ligands, stressing the need for highly targeted approaches into the physiology of mAChR system with selective opto- and chemogenetic methods.

### Radial Arm Maze

The Radial arm maze presents another task used to test spatial cognition, namely working and reference memory, but the procedure may also be adjusted to assess acquisition and memory retrieval (91, 92). This task was used for example in the study of Kay et al. (93), which showed that scopolamine elicits a stronger effect on working memory, while 3,4-methylendioxy-metamphtamine administration affects reference memory more prominently (93). Similar results regarding scopolamine administration had also been reported by Pilcher et al. (91), who compared the effects of scopolamine on working memory, acquisition and memory retrieval, concluding that there was stronger impairment in working memory relative to the other types (91).

This task may also be used for investigating differences in the consequences of acute vs. chronic drug administration, as shown for example by Ortega-Alvaro et al. (94). In their study, the authors found a significant impairment in rats’ performance in the radial arm maze following an acute injection of atypical antipsychotics (olanzapine and clozapine, used in the treatment of schizophrenia) and scopolamine, marked among others by a lower speed of movement. However, when following a chronic drug treatment, the observed deficits were absent, hence hinting at the ability to build a tolerance. The authors also concluded that chronic muscarinic antagonism may exert little or no influence over working memory (94).

One possible drawback of this task was raised in a study of Hodges et al. (95). The authors pointed out that the peripheral effects of scopolamine administration include “dry mouth,” which can lead to disruption of a rat’s ability to eat multiple food pellets and thus decrease their reward value.

### Spatial Alternation Tasks

The natural tendency of rodents to alternate between two choices in successive trials is exploited in a variety of simple T-shaped or Y-shaped mazes. Due to the simplicity of the task, alternation has been employed in the bulk of pharmacological studies using the scopolamine-induced amnesia model. Numerous studies [reviewed in Ref. (96)] have consistently shown that scopolamine treatment disrupts working memory both in discrete (97) and continuous versions of the alternation paradigm (98, 99). A article by Givens and Olton (100) demonstrated that intraseptal injections of scopolamine mimicked the detrimental dose-dependent effect of systemic scopolamine injections, indicating a critical contribution of the medial septal area. Further studies supported the central position of the septohippocampal pathway and revealed a more distributed network including a few other limbic and non-limbic structures (101).

Intraventricular administration of the M1 antagonist pirenzepine exerts similar effects as scopolamine, suggesting that M1 receptors may dominate in mediating spontaneous spatial alternation (102). On the other hand, M2 knockout mice were found to perform worse only under longer (20 s) but not short (5 s) delays in reinforced alternation in a T-maze compared to wild-type controls (103), suggesting a more complex contribution of particular mAChR types. M5 receptors seem to play a role in alternation as well, but the mechanism of action is likely indirect. As M5 receptors are expressed by endothelial cells and control cerebral vasodilatation, M5R−/− mice were found to exhibit a significantly reduced cerebral blood flow in the cerebral cortex, hippocampus, basal ganglia, and thalamus. In consequence, the low blood supply led to impaired long-term potentiation and consequently to a deterioration of spatial alternation (104).

Despite being almost ubiquitous in pharmacological research, the spatial alternation paradigm has some drawbacks. Investigators do not usually configure the maze to enforce animals to use praxis, taxon, or mapping strategies, or any combination of these. Therefore authors cannot report, in contrast to the MWM, whether effects are due to impairment of a particular mode of place navigation. Furthermore, the variability and consistency of results have been disputed, particularly in the spontaneous alternation paradigm. However, this drawback can be counterbalanced by the fact that under some circumstances, spatial alternation has been found to be superb at detecting hippocampal dysfunction (105).

### Active Place Avoidance Tasks

Active place avoidance [(106–117), for review see Ref. (110, 111)] is a behavioral test specifically focusing on a rat’s ability to coordinate two conflicting frames of reference. An animal is placed into a slowly rotating arena where it needs to learn to locate a “to-be-avoided sector,” upon which stepping into it receives a foot-shock. The position of this sector does not change relative to the room frame; i.e., the animal has to actively move to another place in the arena so as not to be carried into the sector. The arena’s surroundings ought to contain distinct extramaze cues for the rats to navigate (110, 112–116).

The first study with scopolamine in this task (117) showed that a deficit induced by scopolamine at doses 1 and 2 mg/kg was not alleviated by intact spatial pretraining. A follow-up study (67) compared the performance of two rat strains obtained from the breeding colony of Institute of Physiology, CAS, Prague (Long-Evans and Wistar) in the MWM and active place avoidance following scopolamine treatment. As already mentioned, whereas in the MWM the disruption in learning and memory was similar, in active place avoidance the Wistar rats exhibited a higher sensitivity to scopolamine than the Long-Evans group (67). In general, active place avoidance tasks are sensitive to antimuscarinic action elicited by scopolamine, yet the effects are strain-specific and also present at relatively higher doses that can also affect procedural aspects. Unfortunately, no active place avoidance results on more selective antagonists, mAChR knockouts or other specific manipulations with the mAChR system are available, indicating the need for future research.

### Barnes Maze

In the Barnes maze, a rat is placed in the center of a circular platform with holes at the edges. An escape cylinder is placed under one of these holes; the animals are trained to locate the position of this cylinder based on distal external cues. The use of odor trails is eliminated by rotating the platform in between trials, and animals presumably use a mapping strategy to locate the target (118).

Evaluations of antimuscarinic agents employing this paradigm are scarce. Consistent with other cognitive mapping taxing tasks, scopolamine was found to impair performance (119). Seeger et al. (103) used this task for investigating changes in cognition and behavior in M2 knock-out mice, reporting a severe impairment in learning, accompanied with decreased short-term and long-term potentiation (103). Another example of the usage of this test is the study by Gawel et al. (120), in which the authors examined the potential of cholinesterase inhibitors (donepezil and rivastigmine) to alleviate ethanol-induced cognitive impairment. The results showed an improvement in both memory retention and cognitive flexibility, the latter being more pronounced for rivastigmine (120).

### Cone-Field Test

The cone-field task represents another experimental paradigm for testing spatial learning and memory. It consists of a dodecagonal field with a number of cones topped with un/baited food cups in the middle and four starting boxes on the borders, from which the animal is released into the field. The ability of the rat to learn and remember the position of the baited cones is assessed. A suggested advantage of this test over tasks like the MWM is that it is based on positive reward learning (whereas the MWM relies on aversive learning). This task was used for example by Van der Staay et al. (121) to investigate the effects of AChE inhibitors (donepezil and metrifonate) on scopolamine-induced learning deficits in rats. The results showed that metrifonate, but not donepezil, was able to alleviate the working memory disruption produced by scopolamine (121). Specific conclusions on the role of mAChRs in this task are impossible due to the limited data.

### Hole-Board Task

In the hole-board task, an animal is placed in a rectangular box with a number of holes in the floor. Some of these are baited with a food reward. An animal is evaluated in its ability to learn and remember the position (using a mapping strategy) of the baited holes as well as the holes it has already visited. Different variations and adaptions of this task have been used. For example, Post et al. (122) published a article on a hole-board paradigm specially designed for mice (COGITAT) and presented its validation as a tool for testing spatial learning and memory *via* a scopolamine-induced performance deficit and its alleviation by metrifonate (122). Regarding the involvement of particular types of receptors, M1 receptors were shown to be important for reference memory (for non-baited holes) in a study evaluating biperiden in pigs (47). On the other hand, M2 receptors were shown to be important for working memory (memory for already-visited holes) in a study using transgenic mice (123).

## General Discussion and Concluding Remarks

The muscarinic system of the brain plays a pivotal role in advanced cognitive processes such as spatial navigation and learning, an extensively studied ability, not only to gain insight into the way humans and animals orient themselves in both familiar and unfamiliar environments, but because spatial memory represents a rodent model of human perceptual memory. Research in this field provides new findings regarding the neurophysiology of higher cognitive processes, as well as pathologies such as those seen in AD and other neurodegenerative diseases, and indicates potential pathways for the therapy and treatment of these conditions.

However, as the muscarinic system is important not only for learning, memory and cognition but also takes parts in other processes such as attention, motivation, sensory perception, and other non-cognitive aspects of behavior, it is no surprise that the blockage of mAChRs also yields a wide range of non-cognitive effects, thus hindering cognition-focused research and complicating interpretations of the effects observed in rodent behavioral experiments. There have been attempts to isolate the purely cognitive effects of muscarinic antagonism from the procedural and motivational aspects, and some have been relatively successful.

One of the more promising ways to study the effects of mAChRs in place navigation lies in the exploitation of local intracerebral administration of antagonists, which ensures no peripheral effects, or the use of specific conditional mutations. Moreover, despite attempts to use more specific muscarininc ligands to eliminate the procedural adverse effect of non-selective antagonists such as scopolamine and atropine, they have often provided ambiguous results. However, Sambeth et al. (24) recently showed that biperiden elicits cognitive deficits extending to the spatial memory domain in humans. It seems that with some caution, a general recommendation of using either non-specific or highly specific antagonists can be provided in conditions with defined place learning strategies having known involvement of the mAChR system.

Nonetheless, the ultimate need and relevance lies in the exploitation of novel techniques such as optogenetics focused on cholinergic neurons, and chemogenetics aimed at cells expressing metabotropic mAChRs. As these methods provide a more precise way to target the mAChR in the CNS, it is conceivable that relatively soon the systemic or even focal application of non-specific antimuscarinic drugs may become a rather obsolete tool for this research. However, the pharmacological development of more specific ligands for mAChRs may yet bring a revival of this traditional neuropharmacology approach. Furthermore, the need for the development of new therapeutics acting on mAChRs will result in an ongoing requirement for testing place navigation as a “prototype” of cognitive functions under the influence of these drugs.

It should also be noted that the choice of a specific behavioral test plays an essential role in the research of cognition, as various tasks examine different aspects of learning and memory (e.g., *praxis* vs. spatial mapping) and may possess higher or lower sensitivity toward the observed phenomenon. Furthermore, not all tasks are hippocampus-dependent, and even among those which are not all employ M1 as a crucial part (Table 1). Careful attention should also be paid to the rodent strain used; for example, albino rats such as the Wistar strain have difficulty learning vision-reliant tasks. Well-planned rodent behavioral studies with carefully thought-out experimental designs will continue to provide a useful tool for research on the muscarinic system and its role in learning and memory.

**Table 1:** Summary of the overall effects (positive, negative, none) of particular groups of antimuscarinergic agents or transgenic manipulations on spatial performance.

	Non-selectives	M1 group antagonists	M1 knockout	M2 group antagonists	M2 knockout
Water maze	Negative (52, 65, 67, 68, 82, 83)	Negative (84, 85)	None (86)	Positive (88)	
Radial arm maze	Negative (91, 93)				
Alternation	Negative (97–99)	Negative (102)			Negative (103)
Active place avoidance	Negative (67, 117)				
Barnes maze	Negative (119, 120)				Negative (103)
Cone field	Negative (121)				
Hole board	Negative (47, 122)				Negative (123)

*M1, M2, muscarinic receptors. Blank cells indicate no data available*.

## Author Contributions

JS, AP, and AS wrote major parts of the manuscript. JS and AS contributed to revisions of the manuscript. AS provided scientific leadership and student supervision. The article is based on the thesis of AP.

## Conflict of Interest Statement

The authors declare that the research was conducted in the absence of any commercial or financial relationships that could be construed as a potential conflict of interest.

## Abbreviations

ACh, acetylcholine; AChE, acetylcholine esterase; AChR, acetylcholine receptors; AD, Alzheimer’s disease; Asn, asparagine; CNS, central nervous system; DMP, delayed matching to position; GPCRs, G-protein-coupled receptors; mAChR, muscarinic acetylcholine receptors; MDMA, 3,4-methylendioxy-metamphtamine; MWM, Morris water maze; nAChR, nicotinic acetylcholine receptors; QNB, quinuclidinyl benzilate.
